# Supplementary material for: Comparative Structure-Property Characterization of Poly(3-Hydroxybutyrate-Co-3-Hydroxyvalerate)s Films under Hydrolytic and Enzymatic Degradation: Finding a Transition Point in 3-Hydroxyvalerate Content
Source: Polymers (Basel). 2020 Mar 24;12(3):728. doi: 10.3390/polym12030728 (PMC7183050; doi:10.3390/polym12030728)
Supplement: Supplementary file 1 [file polymers-12-00728-s001.pdf]

Comparative Structure-Property Characterization of Poly(3-Hydroxybutyrate-Co-3-Hydroxyvalerate)s Films Under Hydrolytic And Enzymatic Degradation: Finding a Transition Point in 3-Hydroxyvalerate Content

Vsevolod A. Zhuikov, Yuliya V. Zhuikova, Tatiana K. Makhina, Vera L. Myshkina, Alexey Rusakov, Alexey Useinov, Vera V. Voinova, Garina A. Bonartseva, Alexandr A. Berlin, Anton P. Bonartsev and Alexey L. Iordanskii

S1. Biosynthesis of Polyhydroxyalkanoates (PHA)

S1.1. Material and methods

For the biosynthesis of polymers, a highly effective strain-producing poly(3-hydroxybutyrate) (PHB) A. *Chroococcum 7B*, a non-symbiotic nitrogen-fixing bacterium, capable of overproducing the polymer (up to 80% of the dry weight of cells) was used [1–5]. The strain was isolated from the wheat rhizosphere (sod-podzolic soil) and was maintained on Ashby medium containing 0.2 g/L $K_2HPO_4{\cdot}3H_2O$, 0.2 g/L MgSO4·7H_2O, 0.2 g/L NaCl, 0.006 g/L $Na_2MoO_4{\cdot}2H_2O$, 5.0 g/L $CaCO_3$, 20 g/L sucrose и 20 g/L agar. All experiments were carried out under laboratory conditions. To achieve high cell productivity, the Azotobacter culture was grown in rocking flasks in a microbiological shaker Innova 43 (New Brunswick Scientific, Edison, NJ, USA) with constant agitation and 30 °C on a Burke medium under conditions of an excessive content of a carbon source in a medium containing 0.4 g/l $MgSO_4{\cdot}7H_2O$, 0.01 g/L $FeSO_4{\cdot}7H_2O$, 0.006 g/L $Na_2MoO_4{\cdot}2H_2O$, 0.5 g/L sodium citrate, 0.1 g/L $CaCl_2$, 1.05 g/L $K_2HPO_4{\cdot}3H_2O$, 0.2 g/L KH_2PO_4 и 17 g/L (50 мМ) sucrose as the main carbon source. For the biosynthesis of the PHB copolymers in the culture medium, the valerian salt was added as an additional carbon source. For the biosynthesis of PHB copolymers, salts of carboxylic acids (propionic, valeric, hexanoic) as additional carbon sources were added to the culture medium. These concentrations and time intervals were chosen to prepare a poly(3-hydroxybutyrate-co-3-hydroxyvalerate) (PHBV) copolymer with different content of 3-hydroxyvalerate (HV) in the chain of the resulting copolymer [1–5]. The production strain was cultured for 72 h. The optical density of the culture medium was monitored by nephelometry. Biosynthesis parameters of copolymers, such as biomass yield (g/L medium) and total polymer content in cells (weight % of dry cell weight), were measured according to previously developed procedures. The process of isolation and purification of the polymer from the biomass of the producer strain included extraction with chloroform, filtration, precipitation with isopropyl alcohol, purification by several dissolution-precipitation cycles, and drying [1–5].

1H-NMR spectra of 1–2% PHB and PHBV solutions in deuterated chloroform were recorded in an MSL-300 (Bruker, Germany) spectrometer at a working frequency of 300 MHz. Chemical shifts were measured relative to the signal of $CDCl_3$ residual protons, 7.20 ppm. The number of accumulations NS = 40. The percent content of elementary HV elements in the PHBV copolymer was calculated according to the ratio of the integral signal intensity from HV methyl group (0.89 ppm) to the sum of integral signal intensities from the methyl groups of HV and hydroxybutyrate (1.27 ppm).

The results of the biosynthesis of the PHBV culture of A. *chroococcum 7B* are shown in Table S1 when grown on a medium with sucrose as the main carbon source and a number of carboxylic acids as an additional carbon source for the synthesis of the copolymer.

Table S1. Biosynthesis of copolymers of sodium phosphate buffer (PBS) with the A. *chroococcum* 7B producer strain in the culture medium with sucrose as the main carbon source and salts of carboxylic acids as additional sources of carbon and precursors for biosynthesis of the copolymers of poly(3-hydroxybutyrate) (PHB). HV = hydroxyvalerate. PEG = polyethylene glycol.

**Substrate**	**Time of addition of the carboxylic acid salt to the culture medium, hours**	**The biomass crop, g/L of medium**	**Polyhydroxyalkanoates (PHA) content in biomass,% of dry cell weight**	**MW of PHA, ×10^6 Da**	**The content of 3OB/3OB in the copolymer, %**
Sucrose, 50 мM	-	5.8 ± 0.6	83.4 ± 3.1	1.70	0
C + 5 мM VA	12	4.4 ± 0.9*	76.2 ± 3.0*	1.29	2.5
C + 10 мM VA	12	4.2 ± 0.9*	73.8 ± 3.7*	1.39	9.0
C + 20 mM VA	9	3.5 ± 0.8*	70.5 ± 3.2*	1.15	17.6
C + 10 мM VA + 20 mM SA	12/0	3.1 ± 1.3*	67.4 ± 4.6*	1.22	5.9
C + 20 мM 4 methylvaleric acid	12	3,4 ± 0,9*	76,7 ± 3,3*	1,30	0,60
C + 20 мM VA+ 150 mM PEG 300	12/0	2,6	49,5	0,219	4,69% - 3-HV<br>0,15% - PEG

* p< 0.05 when compared with the group "Sucrose", n = 8.

1H-NMR spectra of copolymer samples with the maximum inclusion of HV in the PHBV 17 mol. % (with the application of 20 mM valerate) and a minimum inclusion of HV PHBV 2.5% (option with 20 mM propionate), as well as a homopolymer of PHB (sucrose only), were showed in Figure S1. The signal of the methyl group HV was detected at the chemical shift value of 0.89 ppm (Figure S1a,b), whereas in the spectrum of the PHB homopolymer (Figure S1c), this signal was absent.

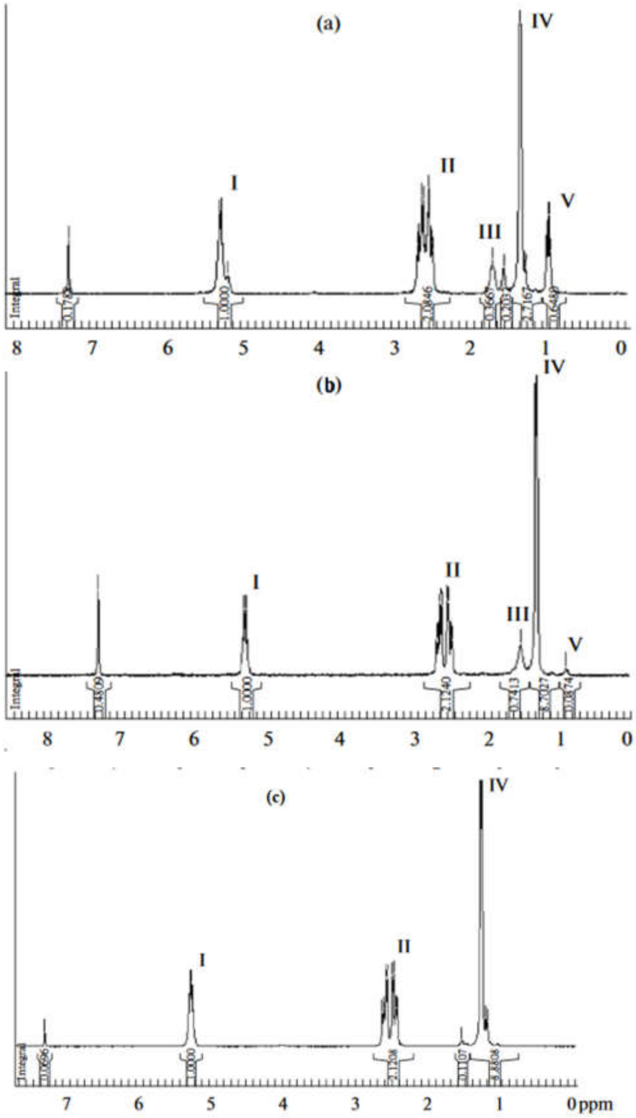

Figure S1. ^{1}H-NMR spectra of a poly(3-hydroxybutyrate-co-3-hydroxyvalerate) (PHBV) copolymer with a content of HV 17% (**a**); HV 2.5% (**b**) and homopolymer PHB (**c**), I – CH(b), II – CH_2(b), III – CH_2(s)HV, IV – CH_3(s)HB, V – CH_3(s) HV, s – side chain; b – polymer backbone.

Thus, PHBV copolymer with different molar percentage incorporation in the HV polymer chain can be obtained by adding to the culture medium valeric acid, propionic acid, and hexanoic acid.

A new PHB4MV copolymer was synthesize by adding the 4-methylvaleric acid to the culture medium as an additional carbon source and the precursor of 3H4MV monomer in the copolymer chain. The incorporation of 3H4MV residues into the synthesized polymer PHB4MV was also confirmed by ^{1}H-NMR. On the ^{1}H-NMR spectrum, the 4-methyl group (e) and the –CH group (g) of the 3H4MV monomer appear as peaks at 0.90 ppm and 1.91, respectively (Figure S2), while the PHB homopolymer and the PHBV copolymer in this region have no signals.

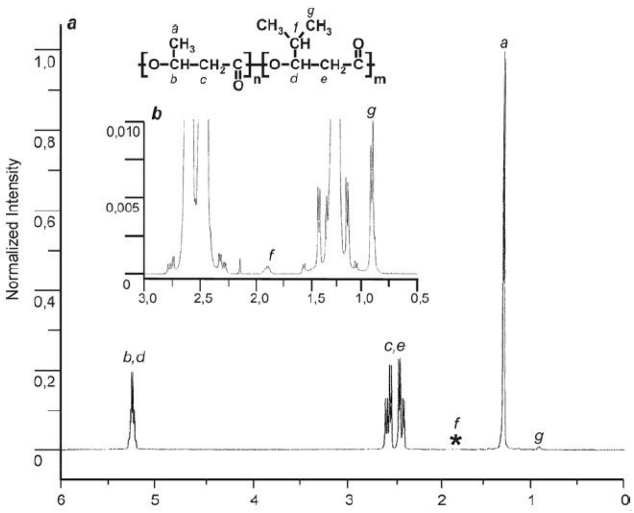

Figure S2. ^{1}H-NMR spectra of a PHBV copolymer with a content of 3-hydroxy-4-methylvalerate (PHB4MV) (a) PHB chain: 1 –CH_3(s), 2 – CH(b), 3 – CH_2(b), PHB-4MV chain (b): 4 – CH_2(s), 5 – CH_3(s), 6 –CH(b), 7 – CH_2(b), s – side chain; b – polymer backbone.

For the synthesis of new copolymers with polyethylene glycol-300, polyethylene glycol (PEG) was used as additives in the culture medium. The addition of these components at a concentration of 150 mM with sucrose also leads to the inclusion of ethylene glycol monomers in the synthesized PHB polymer. The inclusion of monomers was confirmed by ^{1}H-NMR spectroscopy of the newly-synthesized PHB-PEG copolymer [3,4].

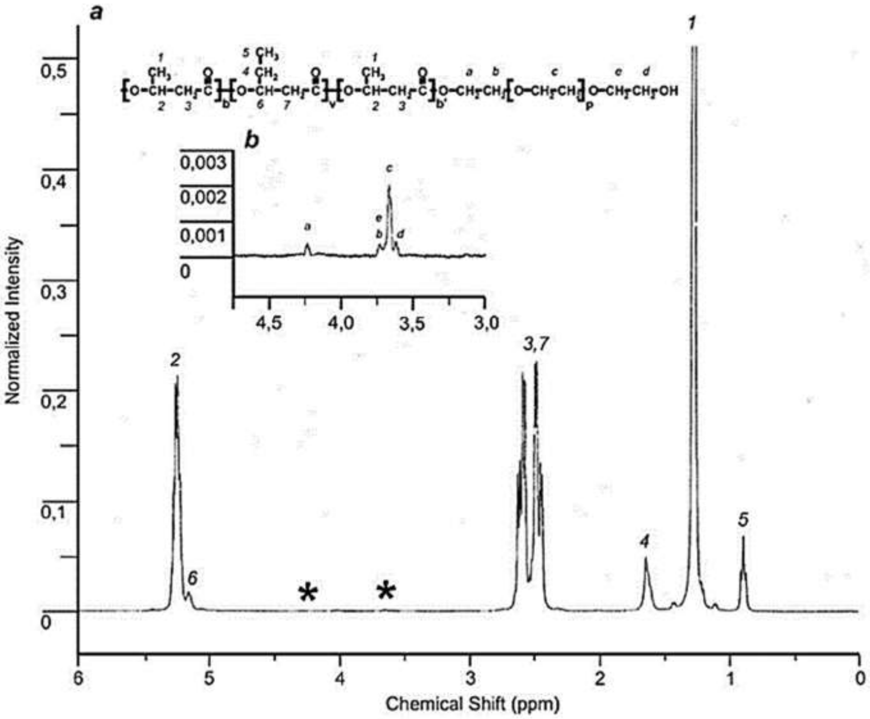

Figure S3. 1H-NMR spectra of a PHBV-PEG: (a) – PHB chain: 1 - CH3(s), 2 - CH(b), 3 - CH2(b), PHBV chain: 4 - CH_2(s), 5 - CH_3(s), 6 - CH(b), 7 - CH_2(b), s – side chain; b – polymer backbone.; enlarged plot of the graph is shown in the inset (b); (b) PEG chain: «a» -O–CH_2 (4.24 ppm), «b» - CH_2 (3.73), «c» - common signal from the middle groups [-O–CH_2–CH_2-] (3.66 ppm), «e» and «d» end groups –CH_2- (3.70 ppm) and –CH_2-OH (3.61 ppm) [3,4].

The melting and crystallization temperatures of the polymers were measured using differential scanning calorimetry. The melting points of both homopolymers and copolymers had a forked peak.

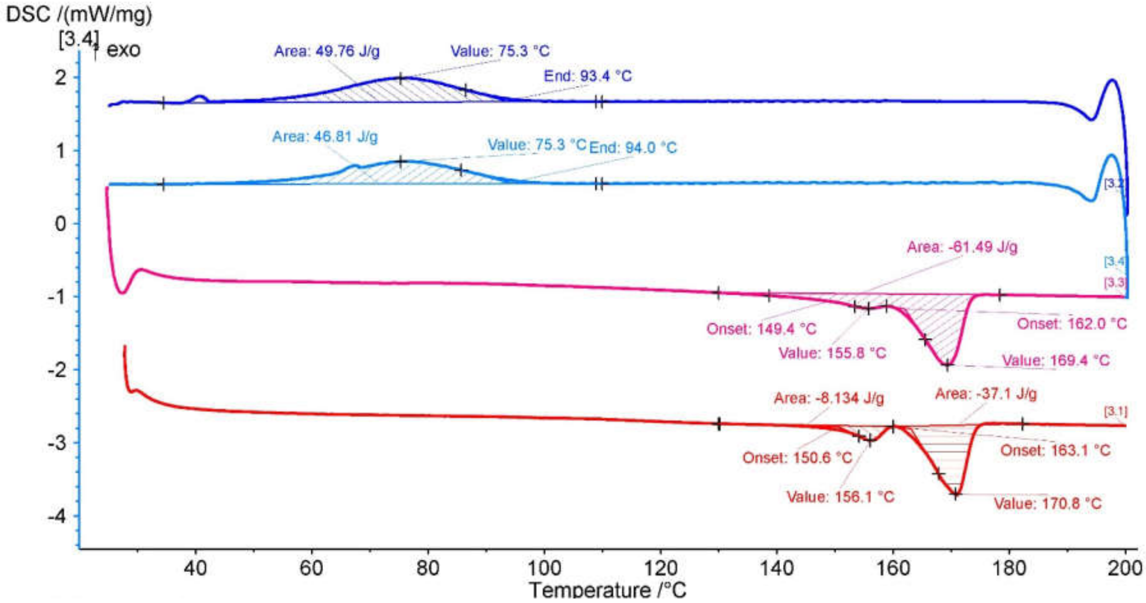

Figure S4. Thermogram of the PHBV copolymer 17.6% 1190 obtained by the DSC method. The thermogram shows two heating curves (red and purple lines) and two cooling curves of the sample (white blue and deep blue lines).

The bifurcated peak in homopolymers is explained by the presence of crystallites of varying degrees of perfection, which may be due to both the thermal history of the sample and the wide molecular weight distribution. In copolymers, the possible appearance of a double peak is explained by the presence of copolymer residues having a lower melting point.

To analyze the synthesized poly(3-hydroxybutyrate), gel permeation chromatography was used. The solvent was chloroform with the addition of 3% vol. methanol. The elution rate was 1 mL/min. The detector is a refractometer. The concentration of the sample is 5 mg/mL. Sample volume −100 μL. Columns—Pl gel 50, 10^3, 10^5 A. Calibration was carried out using polystyrene reference samples with a narrow distribution (less than 1.1).

Table S2. Molecular mass of newly synthesized poly (3-hydroxybutyrate) obtained by various purification methods.

No	$M_w \times 10^3$	$M_n \times 10^3$	M_w/M_n	$M_z \times 10^3$	$Mw \times 10^3$ (viscometry)
1	543.6	332.7	1.63	809.8	554
2	876.2	666.8	1.31	1093.6	881
3	605.8	285.4	2.12	949.8	627

Samples of poly (3-hydroxybutyrate) differed in solvent and isolation method.

According to the table it can be seen that the polymers have a small polydispersity. The data obtained by gel permeation chromatography corresponded to those obtained by the method of viscometry. Therefore, for further study of molecular weight, the method of viscometry was used.

Young's modulus of the films was measured by nanoindentation. The main point of the method is that an indenter with a diamond pyramid at the end is immersed in the sample with a certain force. Further, the load curves (indenter deepening) and unloading curves (indenter output) are taken. The resulting curves are processed and Young's modulus is calculated. The characteristic curve is shown Figure S5.

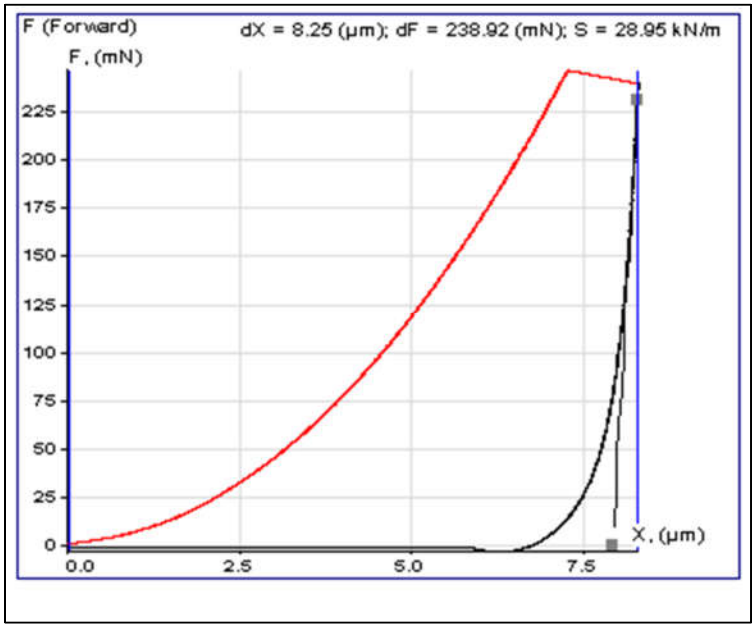

Figure S5. Image of a characteristic curve in the coordinates of the dependence of the applied force on the indenter displacement obtained using the nanoindentation method.

The characteristic image of a drop of water on the surface of films of synthesized polymers is presented below. This image is taken after calculating the contact angle.

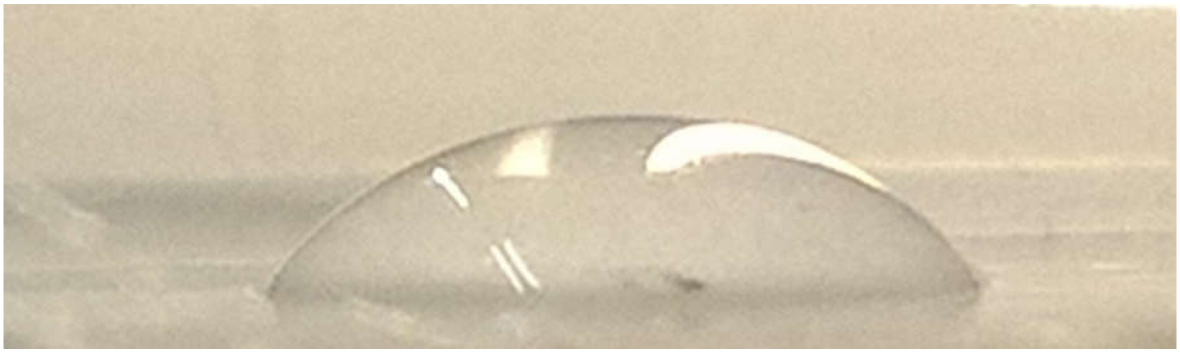

Figure S6. The image of a drop of water on the surface of a polymer film.

S2. Lipase Concentration Selection

In the literature devoted to the problem of PHB biodegradation, own bacterial enzymes are widely used as an enzyme decomposing a polymer [6–8]. However, the decomposition of the polymer will be affected not only by the enzyme but also by the buffer solution in which this enzyme is dissolved [1]. Therefore, the choice of a buffer solution in which polymer degradation will take place is an important task. To compare the biodegradation rate of the polymer, two buffer solutions were selected: 0.01 M sodium phosphate buffer (PBS) and a buffer simulating blood plasma (SBF), as well as various concentrations (0; 0.1; 0.25; 1 mg/mL) of pork pancreatic lipase in these solutions. All solutions had a pH of 7.4. PHB films with a molecular weight of 105 kDa were placed in these solutions. The polymer films were stored at a temperature of 37 °C for a month.

Figure S7 shows diagrams of changes in the weight of PHB films after 1 week and a month of biodegradation under the action of pancreatic lipase.

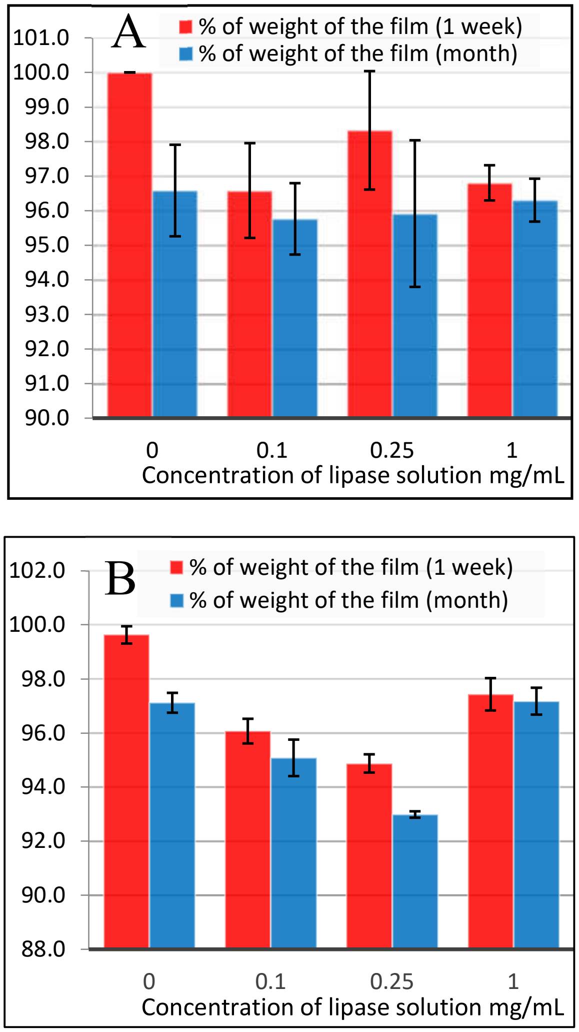

Figure S7. Diagrams of changes in the weight of plates in (**A**) a buffer simulating blood plasma (SBF) and in (**B**) phosphate buffer (PSB) depending on the concentration of pancreatic lipase for a week and a month.

According to the decrease in the wight of PHB films during degradation in both buffer solutions, it can be said that these solutions do not interfere with polymer degradation; however, a lipase solution in sodium phosphate buffer has a greater effect on polymer degradation. After a month, a solution with a lipase concentration of 0.25 mg/mL had the strongest effect on the polymer film, whose wight decreased by 7% from the original values. At lipase concentrations of 0.1 mg/mL and 1 mg/mL, the weight loss of the films averaged 4.7% per month in sodium phosphate buffer and 4% in a buffer simulating blood plasma. This partly confirms the results of [10], in which blood plasma had a negligible effect on polyhydroxybutyrate samples. The effect of lipase concentration can be explained by the fact that at 1 mg/mL the process of substrate inhibition occurs, similar to that described for PHB-depolymerase. At a high concentration of the enzyme, the degradation rate decreases due to the blocking of access of the catalytic domain of the enzyme to polymer molecules [11]. At a concentration of 0.1 mg/mL, not the entire polymer surface is occupied by enzyme molecules; therefore, the degradation rate does not reach its peak. Based on the results obtained, a concentration of 0.25 mg/mL is optimal for experiments on the degradation of PHB, and this concentration of lipase corresponds to the concentration of pancreatic lipase in the human body [12].

S3. Changes in the Surface Morphology of Polyhydroxyalkanoate Films

To describe the morphological changes induced by enzymatic degradation, the films were studied by AFM. The lamellar structure is easily distinguished by phase imaging, so this method was applied. Three types of morphological changes were observed: the emergence of new lamellar structures, fragmentation of lamellar structures, and the disappearance of lamellar structures

The first effect of decomposition—the appearance of new lamellar structures (Figure S8). The arrow shows an extended structure, which can be interpreted as a single lamella (Figure S8A). After treatment with a lipase solution, it becomes more visible, and another lamella appears to the right of it (Figure S8B). These changes in the morphology of the film explained as the stack of lamellas is coated with a thin layer of an amorphous polymer. Lipase, which had adsorbed on the surface of the film, begins to decompose amorphous polymer, exposing the crystalline structures under them.

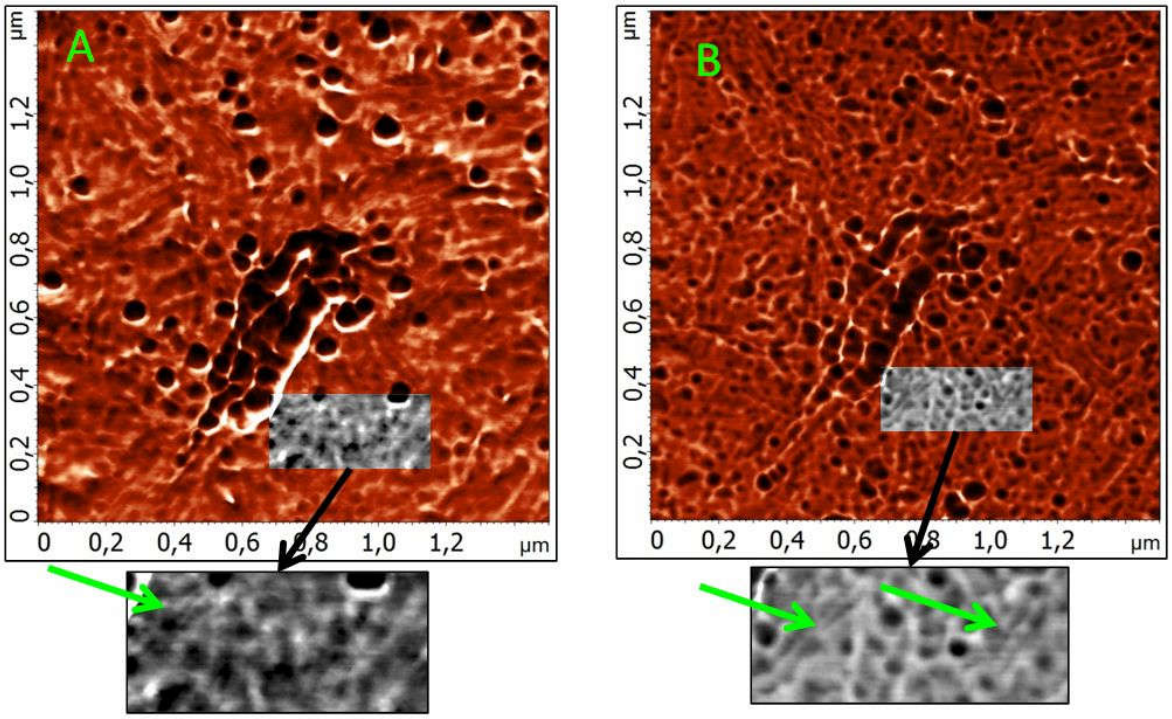

Figure S8. The phase images of PHB film before (**A**) and after (**B**) hydrolysis in lipase solution. Arrows indicate the new lamellas.

The second change in the morphology - the fragmentation of lamellar structures. Figure S9A shows an image of PHB film before enzymatic hydrolysis. The selected area contains a system of parallel strips, interpreted as a stack of lamellae. After treatment with lipase (Figure S9B), lamellae are fragmented. Before enzymatic degradation, lamellae length in the stack was 300 ± 20 nm; after the degradation, the length of the fragments had decreased to 80 ± 10 nm. The reason for that is probably the defects in the crystal structure. Lipase attacks such defects, which result in the disintegration of lamellae into smaller pieces.

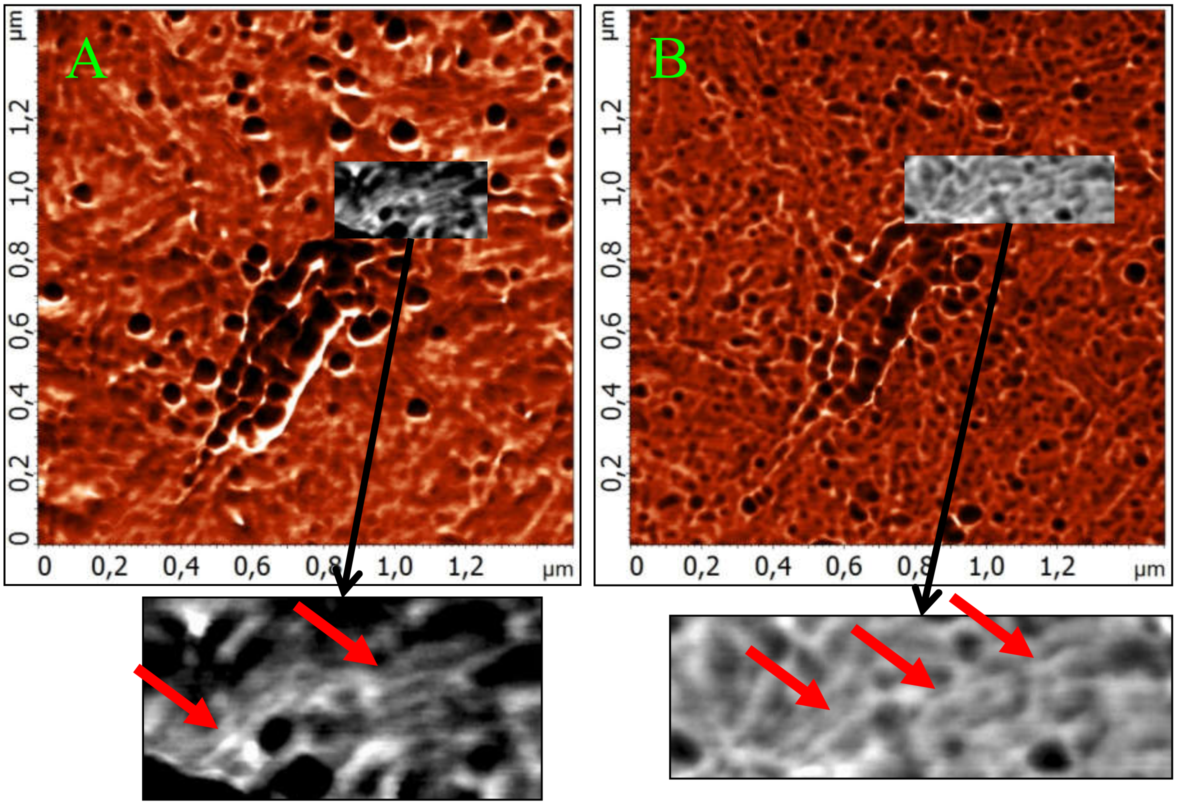

Figure S9. The phase images of PHB film before hydrolysis in lipase solution (**A**) and after (**B**). Arrows indicate the fragmentation of lamellae.

The third morphological change is the disappearance of lamellar structures. Figure S10 shows the PHB film images before and after lipase treatment. A system of parallel strips structure interpreted by us as a stack of lamellae was found on a PHB film before treatment with lipase. After hydrolysis in the lipase solution, this structure has changed dramatically: most of the lamellae have disappeared, and the remaining were highly fragmented (Figure S10). The reason for the disappearance of lamellae is the same, as in the situation with fragmentation of lamellae—there are defects in lamellae, but in this case, their number is much greater, allowing the lipase to almost completely destroy lamellae stack during the experiment [13].

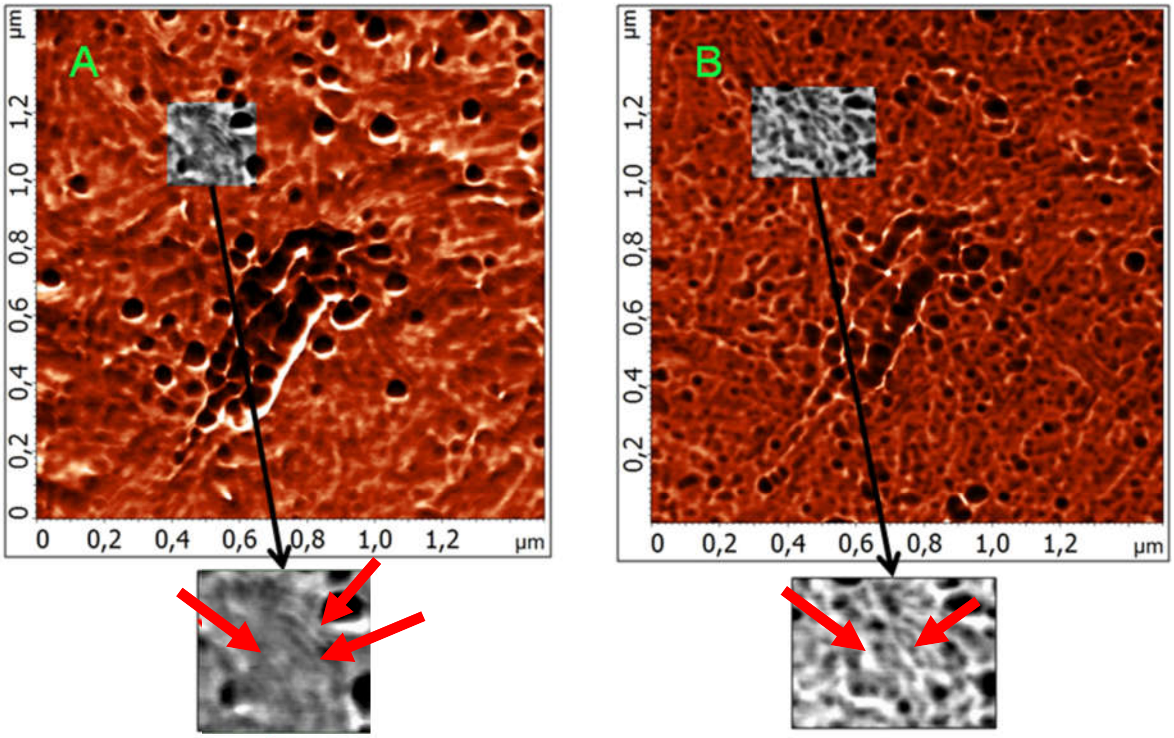

Figure S10. The phase images of PHB film before (**A**) and after (**B**) hydrolysis in lipase solution. Arrows indicate the disappearance of lamellar structures.

It is important to note that changes in a lamellar structure during the process of degradation are typical for other semicrystalline polymers (for example, for polymers, such as polylactide) [14].

References

1. Chen, G.-Q.; Wu, Q. The application of polyhydroxyalkanoates as tissue engineering materials. *Biomaterials* **2005**, *33*, 6565–78.
2. Bonartsev, A.; Yakovlev, S.; Boskhomdzhiev, A.; Zharkova, I.; Bagrov, D.; Myshkina, V.; Mahina, T.; Kharitonova, E.; Samsonova, O.; Zernov, A.; et al. The Terpolymer Produced by Azotobacter Chroococcum 7B: Effect of Surface Properties on Cell Attachment. *PLoS ONE* **2013**, *8*, e57200.
3. Bonartsev, A.P.; Bonartseva, G.A.; Myshkina, V.L.; Voinova, V.V.; Mahina, T.K.; Zharkova, I.I.; Yakovlev, S.G.; Zernov, A.L.; Ivanova, E.V.; Akoulina, E.A.; et al. Biosynthesis of poly(3-hydroxybutyrateco- 3-hydroxy-4-methylvalerate) by Strain Azotobacter chroococcum 7B. *Acta Naturae* **2016**, *3*, 77–87.
4. Myshkina, V.L.l Ivanov, E.A.; Nikolaeva, D.A.; Makhina, T.K.; Bonartsev, A.P.; Filatova, E.V.; Ruzhitsky, A.O.; Bonartseva, G.A. Biosynthesis of poly(3-hydroxybutyrate-co-3-hydroxyvalerate) copolymer by Azotobacter chroococcum strain 7B. *Appl. Biochem. Microbiol.* **2010**, *46*, 289–296.
5. Myshkina, V.L.; Nikolaeva, D.A.; Makhina, T.K.; Bonartsev, A.P.; Bonartseva, G.A. Effect of growth conditions on the molecular weight of poly (3-hydroxybutyrate) produced by Azotobacter chroococcum 7B. *Appl. Biochem. Microbiol.* **2008**, *44*, 482–486.
6. Doi, Y.; Kanesawa, Y.; Kunioka, M.; Saito, T. Biodegradation of microbial copolyesters: Poly(3-hydroxybutyrate-co-3-hydroxyvalerate) and poly(3-hydroxybutyrate-co-4-hydroxybutyrate). *Macromolecules* **1990**, *1*, 26–31.
7. Doi, Y. Microbial synthesis, physical properties, and biodegradability of polyhydroxyalkanoates. Macromolecular *Symposia* **1995**, *1*, 585–599.
8. Numata, K.; Yamashita, K.; Fujita, M.; Tsuge, T.; Kasuya, K.I.; Iwata, T.; Doi, Y.; Abe, H. Adsorption and hydrolysis reactions of poly(hydroxybutyric acid) depolymerases secreted from Ralstonia pickettii T1 and Penicillium funiculosum onto poly[(R)-3-hydroxybutyric acid]. *Biomacromolecules* **2007**, *7*, 2276–2281.

9. Yamashita, K.; Funato, T.; Suzuki, Y.; Teramachi, S.; Doi, Y. Characteristic Interactions between Poly(hydroxybutyrate) Depolymerase and Poly[(R)-3-hydroxybutyrate] Film Studied by a Quartz Crystal Microbalance. *Macromol. Biosci.* **2003**, *11*, 694–702.
10. Bonartsev, A.P.; Myshkina, V.L.; Nikolaeva, D.A.; Furina, E.K.; Makhina, T.A. Biosynthesis, biodegradation, and application of poly (3- hydroxybutyrate) and its copolymers-natural polyesters produced by diazotrophic bacteria. *Commun. Curr. Res. Educ. Top. Trends Appl. Microbiol.* **2007**, *1*, 295–307.
11. Carrière, F.; Renou, C.; Lopez, V.; Caro, J. De; Ferrato, F.; Lengsfeld, H.; Caro, A. De; Laugier, R.; Verger, R. The specific activities of human digestive lipases measured from the in vivo and in vitro lipolysis of test meals. *Gastroenterology* **2000**, *4*, 949–960.
12. Zhuikov,V.A.; Bonartsev, A.P.; Bagrov, D.V.; Yakovlev, S.G.; Myshkina, V.L.; Makhina, T.K.; Bessonov, I.V.; Kopitsyna, M.N.; Morozov, A.S.; Rusakov, A.A.; Useinov, A.S.; et al. Mechanics and surface ultrastructure changes of poly(3-hydroxybutyrate) films during enzymatic degradation in pancreatic lipase solution. *Mol. Cryst. Liq. Cryst.* **2017**, *1*, 236–243.
13. Kikkawa, Y.; Murase, T.; Abe, H.; Iwata, T.; Inoue, Y.; Doi, Y. Real-time enzymatic degradation study of poly[(R)-3-hydroxybutyric acid] copolymer thin film by atomic force microscopy in buffer solution. *Macromol. Biosci.* **2002**, *2*, 189–194.
14. Zong, X.; Ran, S.; Kim, K.-S.; Fang, D.; Hsiao, B.S.; Chu, B. Structure and Morphology Changes during in Vitro Degradation of Electrospun Poly(glycolide-co-lactide) Nanofiber Membrane. *Biomacromolecules* **2003**, *4*, 416–423.
